# An Unguided Web-Based Resilience Training Programme for NHS Keyworkers During the COVID-19 Pandemic: a Usability Study

**DOI:** 10.1007/s41347-021-00225-3

**Published:** 2022-03-17

**Authors:** Matthew R. Bennion, Felicity Baker, Joanna Burrell

**Affiliations:** 1 Department of Computer Science, The University of Sheffield, Sheffield, UK; 2 Department of Psychology, The University of Sheffield, Sheffield, UK; 3 Ultimate Resilience Ltd, Nottingham, UK

## Introduction

In the UK, 12.8 million working days were lost due to work-related stress during the period 2018/2019 (Health & Safety Executive, 2020) with just over 0.6 million workers suffering from work-related stress, anxiety, or depression. These figures are typically higher for staff working in healthcare settings. Emerging research on the impact of the COVID-19 pandemic on health workers’ mental health shows increases in mental health problems and psychological distress (Garciá-Fernández et al., 2020; Spoorthy, 2020), along with low levels of resilience (Mosheva et al., 2020; Roberts et al., 2021).

Providing support to staff in a structured manner is a requirement of the NHS Constitution. Lessons learned from the COVID-19 pandemic, along with an understanding of how previous pandemics and disasters have affected healthcare systems, have highlighted the need for greater focus on support and resilience in the nursing workforce (Duncan, 2020). Resilience is important for all individuals to cope with normal daily life stressors and is particularly important for NHS staff who are often working in highly stressful situations. These stressors have been exacerbated by the COVID-19 pandemic, highlighting the need for NHS Employers to support staff to build resilience to remain healthy, both physically and mentally, and to ensure job retention (Yılmaz, 2017).

Resilience can be defined as ‘the role of mental processes and behaviour in promoting personal assets and protecting an individual from the potential negative effect of stressors’ (Fletcher & Sarkar, 2012). Resilience can be viewed as a set of key skills or personal characteristics that allow improved coping and adaptation through challenge or threat. Resilience is a dynamic process that can be learned, leading to improvements in mental health and wellbeing, improved social support, self-efficacy, and coping. The consequences of such improvements are that staff are better able to adapt to pressures and demands in the workplace and across all areas of their lives (Helmreich et al., 2017).

When considering how to make resilience training readily available to large numbers of busy staff, such as those working in NHS services, it is important to consider novel approaches. Web-based training, including mobile apps, has seen a rise in popularity for both health and mental wellbeing (Bakker et al., 2016; Bennion et al., 2019). This medium has the potential to offer training in resilience to large numbers of people whilst overcoming barriers such as stigma, time, and acceptability, and can be integrated easily into a wider organisational wellbeing strategy. Indeed, digital resilience training has been found to benefit other groups such as students and first responders (Galante et al., 2018; Heyen et al., 2021; Rose et al., 2013). However, at the time of writing, no studies of a web-based resilience training aimed specifically at NHS keyworkers were identified.

### Objectives

The aim of this formative usability study was to assess the acceptability and user perceptions of a web-based resilience training programme created for NHS keyworkers. We hypothesized that the training would be both acceptable and perceived as useable. The study also sought to assess the impact of this training programme on keyworkers’ knowledge of stress and resilience. We hypothesized that the training would result in an increase in perceived knowledge of both stress and resilience.

## Methods

The study was administered by The NIHR Trauma Management MedTech Co-operative based at University Hospitals Birmingham NHS Foundation Trust (UHB) and involved progressing through three distinct stages: baseline measures, web-based resilience training, and post-training measures. The training was hosted on the MedTech website and made available to participants via a web link. The approximate time required for a participant to complete the measures and the training was estimated at 45 min.

### Participants

Participants were self-selected NHS keyworkers, both clinical and non-clinical, recruited through electronic advertisement to clinical departments across UHB and through the circulation of a web-based and paper flyer to staff forums and notice boards across the Trust. To take part, participants were provided with web links and passwords to access the study. Consent was acknowledged by completion of the baseline measures. All data were collected and anonymised. Anonymised email addresses were used to match baseline- and post-measures and ensure participants did not complete the study twice.

### Web-Based Resilience Training Programme

The content of the web-based training drew on the Skills-based Model of Personal Resilience (Baker et al., 2021) and included a selection of evidence-based skills and exercises to regulate distress emotions and build positive emotions, such as slow rhythmic breathing and mindfulness practice.

Bennion et al (2019) highlight four key indicators of quality drawn from effective digital psychotherapy approaches. These include clinician involvement, academic involvement, research, or other evidence and use of specific psychological approach or theory. The intervention followed these recommendations, drawing on academic and clinical theory (Baker et al., 2021), and involving clinicians, academics, and computer scientists in its development to ensure greater quality and effectiveness.

The web-based training was published using Articulate Storyline and accessed via a web browser. It consisted of both written and spoken content on a series of slides, short videos, and experiential exercises which could be moved through at participants’ own pace using ‘previous’ and ‘next’ buttons. The estimated time to complete the training was 20 min.

### Baseline Measures

At baseline, information on sample characteristics was collected digitally including gender, age (years), years of experience in healthcare, current role, and current work area. Participants were also asked to rate their knowledge of stress and resilience on two individual 5-point Likert scales from 1 (very limited) to 5 (very good).

### Post-Training Measures

Post-training measures were completed digitally. Participants were asked to repeat the measure of their knowledge of stress and resilience. They were also asked to complete standardized measures of user experience and usability. These were System Usability Scale (SUS, Brooke, 1996) and Usability Metric for User Experience (UMUX, Finstad, 2010). Both measures have been shown to be reliable, effective measures of usability, with support to show that scores from each correlate well when used in conjunction (Lewis et al., 2015). SUS includes 10 items of measurement on a 5-point Likert scale from 1 (strongly disagree) to 5 (strongly agree). Once completed, this scale produces a single score which indicates the overall usability of the product (between 0 and 100); the higher the score, the better participant perception of product usability. Research deems an average SUS score of 68 to be acceptable (Brooke, 1996). Studies by Bangor et al. (2009) and Brooke (2013) which examined over 1000 SUS scores from different applications and technologies have shown a SUS score of 72 to be good. UMUX includes 4 items of measurement on a 7-point Likert scale from 1 (strongly disagree) to 7 (strongly agree). This produces a single score indicating overall usability (between 0 and 100); higher scores indicate better product usability (Finstad, 2010).

In addition, participants were asked to provide general feedback on the content and process of the web-based training including rating whether the training had enough written, spoken, and interactive content; whether the training was an acceptable pace, easy to understand, engaging, informative, and visually appealing; whether exercises were an acceptable length and easy to follow, and whether they would use the exercise again in future. All questions were given on an individual 5-point Likert scale from 1 (strongly disagree) to 5 (strongly agree).

### Statistical Analysis

Data were analysed using IBM SPSS for Microsoft Windows (version 26). The primary measures for the study were knowledge of stress and resilience. A descriptive analysis was performed to report means and standard deviations of the continuous variables and frequency distribution of the categorical variables. Wilcoxon signed-rank tests were then carried out to test whether there was statistically significant change between baseline and post measures of stress and resilience knowledge.

## Results

### Sample Characteristics

Age of participants ranged from 22 to 62 years, with a median of 41.50. The sample comprised of 76.9% (20/26) females and 23.1% (6/26) males; 65.4% (17/26) clinical and 34.6% (9/26) non-clinical staff and duration of experience from 1 to 40 years, with a mean of 13.92 (SD 9.70). A total of 26 participants completed baseline measures and 24 completed the post-training measures. Those that completed both sets of measures had a median age of 42.00 years; 75% (18/24) were female, and 25% (6/24) were male. Dropout, defined as the number of participants who did not complete both sets of measures, was 7.7% (2/26).

### Knowledge of Stress and Resilience

There was a statistically significant median increase in participants’ perceived knowledge of stress when comparing baseline to post (*z* =  − 3.051; *P* = 0.002). There was also a statistically significant median increase in participants’ perceived knowledge of resilience when comparing baseline to post (*z* =  − 3.695; *P* < 0.001).

### Usability and User Experience

The system usability score for the training was above the acceptable cut-off point of 68, with a mean of 76.15 (SD 11.84). The Usability Metric for User Experience had a mean of 77.77 (SD 14.25). Overall, the web-based resilience training programme had a high user experience rating (see Table 1).

**Table 1:** Mean (SD) for measures at baseline and post-training

Outcome measures	Completers (*n* = 24), mean (SD)
**Perceived stress knowledge**	
Baseline	3.75 (0.74)
Post-training	4.21 (0.59)
**Perceived resilience knowledge**	
Baseline	2.92 (0.78)
Post-training	4.08 (0.65)
**System usability scale**	
Post-training	76.15 (11.84)
**Usability metric for user experience**	
Post-training	77.77 (14.25)

### General Feedback

**Fig. 1:**
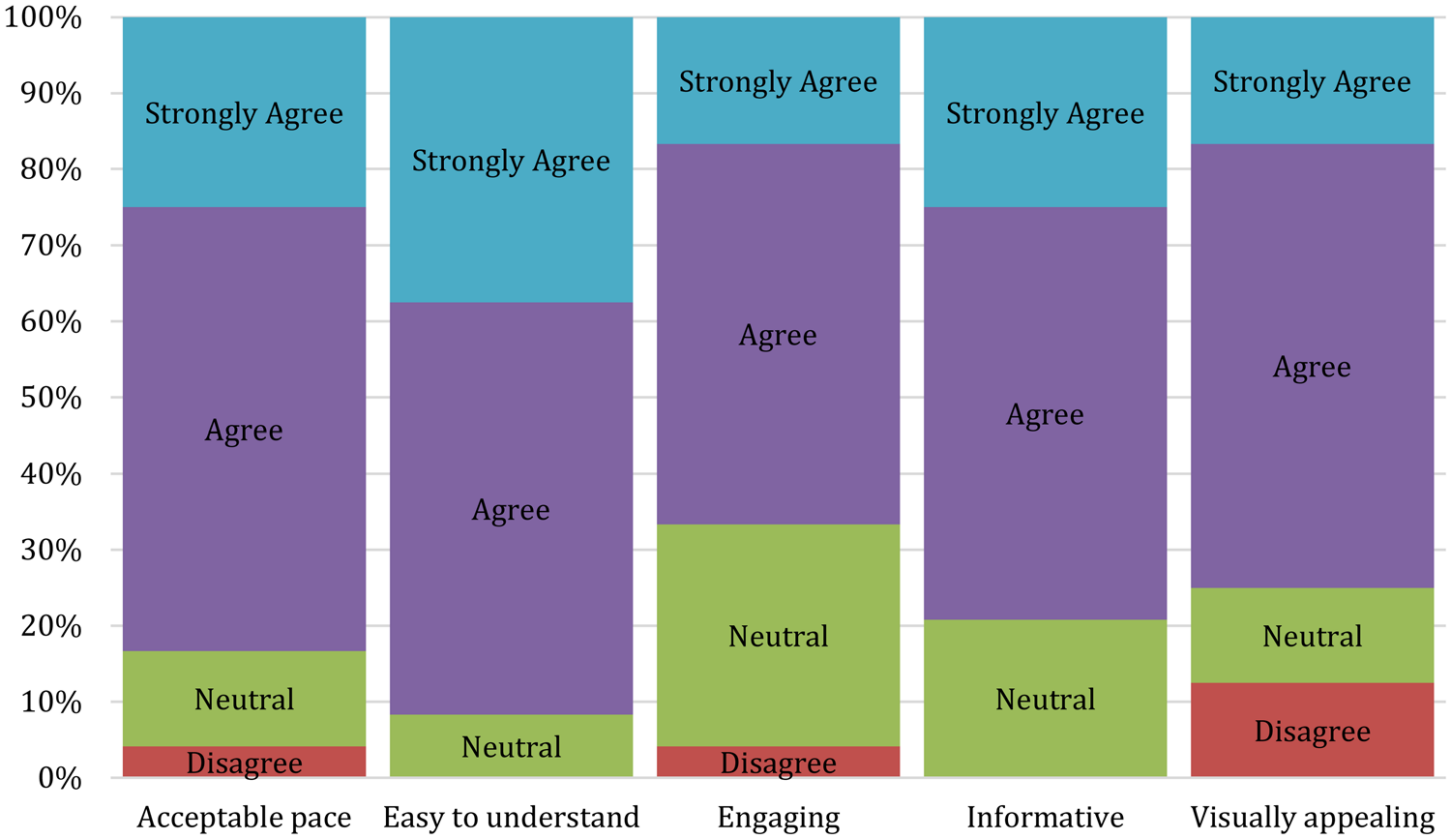
User experience question results

Participants generally agreed there was enough written and spoken content within the training (20/24 agreed/strongly agreed for each these measures). A total of 15/24 felt there were enough interactive exercises (15/24 agreed/strongly agreed), although a significant 7/24 disagreed, suggesting a few extra interactive exercises might be worth adding. The majority of participants either agreed or strongly agreed (ranging from 16/24 to 22/24) that the training was at an acceptable pace, easy to understand, engaging, informative, and visually appealing (see Fig. 1).

## Discussion

The first aim of the study was to assess the acceptability and usability of the training. The results indicated that participants found the training programme both acceptable and usable. The average SUS score for the training was 76 which is deemed good, and the average UMUX score for the training was 78, indicating the training was perceived to align well with the ISO definition of usability (International Organization for Standardization, 2018).

The second aim of the study was to investigate if the training impacted on NHS keyworkers’ perceived knowledge of stress and resilience. There were significant median increases in perceived stress and resilience knowledge, indicating that participants increased their knowledge through participating in the training. Whilst this study did not aim to evaluate outcomes, the reported increase in knowledge of stress and resilience highlights the potential for this form of intervention to raise awareness and contribute to greater resilience. This result is consistent with other studies that have found improvements in mental health and wellbeing using computerised and/or web-based approaches (Bakker et al., 2016; Blake et al., 2020; Rich et al., 2020).

Whilst there have been positive steps towards digital delivery of training generally within the NHS, staff wellbeing and resilience training are presently limited on e-learning platforms. The NHS response to COVID-19 which makes use of Heath Education England’s e-Learning for Healthcare platform (NHS England and NHS Improvement, 2020) offers opportunity for digital delivery of resilience training to NHS keyworkers providing accessible staff training to high numbers of participants. If found to be effective, this type of digital training could be used to supplement standard face-to-face delivery of training of this nature.

This study focused its evaluation on perceived usability, not obtained through lab-based observations, and this may limit its representativeness of true user experience. Further, carrying out a heuristic evaluation of the training to detect usability problems would have been useful to include, but pandemic restrictions made this problematic to implement.

The addition of qualitative measures would provide deeper insight into what NHS keyworkers felt about the training. A more robust method of measuring knowledge acquisition and retention would have further benefitted this study. In addition, whilst the design of the study reflects some of the key parameters required for an effective online survey, the study could be further improved by more closely adhering to the CHERRIES checklist (Eysenbach, 2004).

To the best of our knowledge, this is the first study to apply accepted models of system usability (e.g. International Organization for Standardization, 2018) in the evaluation of a web-based resilience training programme intended to help NHS keyworkers learn and practice resilience skills.

Whilst further research is needed to assess the impact of including more interactive exercises to heighten engagement, to evaluate resilience outcomes and to develop an effective prototype, the data presented here broadly supports the potential use of a web-based programme for training NHS keyworkers in resilience skills.

## Declarations

### Disclaimer

The views expressed are those of the authors and not necessarily those of the NHS, the NIHR, or the Department of Health and Social Care.
